# (5-Bromo-2-meth­oxy­phen­yl)(4-ethyl­cyclo­hex­yl)methanone

**DOI:** 10.1107/S1600536811013687

**Published:** 2011-04-16

**Authors:** Ling Wang, Ziqian Chang, Chunlei Ding, Hua Shao, Jianzi Sun

**Affiliations:** a Pharmacy Department of the Second Artillery General Hospital, Beijing 100088, People’s Republic of China; b Tianjin Key Laboratory of Molecular Design and Drug Discovery, Tianjin Institute of Pharmaceutical Research, Tianjin, 300193, People’s Republic of China

## Abstract

In the title compound, C_16_H_21_BrO_2_, the cyclo­hexane ring is in a chair conformation and its least-squares plane is at an angle of 61.3 (9)° to the benzene ring. The crystal packing is stabilized by weak π–π stacking inter­actions [centroid–centroid distance = 3.697 (9) Å] between the bromo­meth­oxy­phenyl rings of neighbouring mol­ecules.

## Related literature

For the anti­hyperglycemic activity of SGLT2 inhibitors, see: Gao *et al.* (2010 ▶); Meng *et al.* (2008 ▶); Shao *et al.* (2010 ▶). For bond-length data, see: Allen *et al.* (1987 ▶).
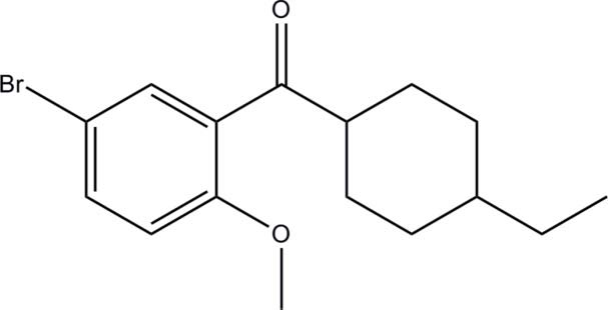


## Experimental

### 

#### Crystal data

C_16_H_21_BrO_2_
*M* *_r_* = 325.24
Monoclinic, 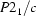

*a* = 14.204 (3) Å
*b* = 11.276 (2) Å
*c* = 9.604 (2) Å
β = 102.329 (4)°
*V* = 1502.8 (6) Å^3^
*Z* = 4
Mo *K*α radiation
μ = 2.73 mm^−1^
*T* = 113 K
0.26 × 0.22 × 0.20 mm

#### Data collection

Rigaku Saturn CCD area-detector diffractometer
Absorption correction: multi-scan (*CrystalClear*; Rigaku, 2007 ▶) *T*_min_ = 0.537, *T*_max_ = 0.611
13836 measured reflections
3588 independent reflections
2581 reflections with *I* > 2σ(*I*)
*R*_int_ = 0.033

#### Refinement

*R*[*F* ^2^ > 2σ(*F* ^2^)] = 0.024
*wR*(*F* ^2^) = 0.058
*S* = 1.04
3588 reflections
174 parameters
H-atom parameters constrained
Δρ_max_ = 0.52 e Å^−3^
Δρ_min_ = −0.28 e Å^−3^

### 

Data collection: *CrystalClear* (Rigaku, 2007 ▶); cell refinement: *CrystalClear*; data reduction: *CrystalClear*; program(s) used to solve structure: *SHELXS97* (Sheldrick, 2008 ▶); program(s) used to refine structure: *SHELXL97* (Sheldrick, 2008 ▶); molecular graphics: *SHELXTL* (Sheldrick, 2008 ▶); software used to prepare material for publication: *SHELXTL*.

## Supplementary Material

Crystal structure: contains datablocks I, global. DOI: 10.1107/S1600536811013687/bx2347sup1.cif

Structure factors: contains datablocks I. DOI: 10.1107/S1600536811013687/bx2347Isup2.hkl

Additional supplementary materials:  crystallographic information; 3D view; checkCIF report

## Figures and Tables

**Fig. 1.**
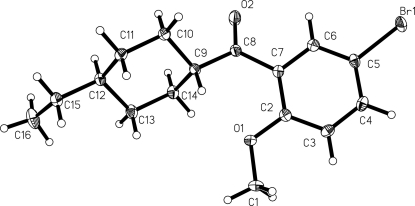
The molecular structure of (I), with atom labels and 40% probability displacement ellipsoids for non-H atoms.

## supplementary crystallographic information

### Comment

SGLT2 inhibitors are a class of promising anti-hyperglycemic agents, and a variety of SGLT2 inhibitors are now in clinical trials (Meng *et al.*, 2008). The title compound was a crucial intermediate, the aglycon of the C-glucoside SGLT2 inhibitors, for the synthesis of novel C-glucoside SGLT2 inhibitors during the development of our own SGLT2 inhibitors (Gao *et al.*, 2010; Shao *et al.*, 2010). The title compound, C_16_H_21_BrO_2_, bond lengths are normal (Allen *et al.*, 1987). The C═O bond of the title compound, C_16_H_21_BrO_2_, is non-coplanar with the benzene ring. The cyclohexane ring is in the chair conformation and its least-squares plane is at an angle of 61.3 (9)° to the benzene ring. No classic hydrogen bonds were found, the crystal packing is stabilized by one weak π–π stacking interaction [centroid-to-centroid distance = 3.697 (9) Å, *Cg*1 is centroid of benzene ring (C2—C7), Symmetry code:1 - *x*, -*y*, 1 - *z*].

### Experimental

A dried 100-ml round-bottomed flask was charged with 1.75 g (10 mmol) of *trans*-4-ethylcyclohexanecarboxylic acid chloride, 1.87 g (10 mmol) of 4-bromoanisole and 20 ml of dried dichloromethane, and the mixture was stirred on an ice-water bath, followed by addition of 1.33 g (10 mmol) of anhydrous aluminium chloride in a portion wise manner. After addition, the reaction mixture was stirred at room temperature overnight and poured into 300 ml of ice-water. The mixture thus formed was extracted with three 50-ml portions of dichloromethane, and the combined exacts were washed successively with 1% hydrochloric acid and saturated brine, dried over sodium sulfate and evaporated on a rotary evaporator to afford the crude title compound. Pure title compound was obtained by column chromatography. Crystals suitable for X-ray diffraction were obtained through slow evaporation of a solution of the pure title compound in dichloromethane/petroleum ether  mixture (1/30 by volume).

### Refinement

H atoms were positioned geometrically and refined using a riding model, with C—H = 0.95 to 1.00 Å; with *U*_iso_(H) = 1.2 times *U*_eq_(C) and 1.5 times *U*_eq_(C)for methyl H atoms.

### Crystal data

**Table:**

C_16_H_21_BrO_2_	*F*(000) = 672
*M**_r_* = 325.24	*D*_x_ = 1.438 Mg m^−^^3^
Monoclinic, *P*2_1_/*c*	Mo *K*α radiation, λ = 0.71073 Å
Hall symbol: -P 2ybc	Cell parameters from 5413 reflections
*a* = 14.204 (3) Å	θ = 2.2–27.9°
*b* = 11.276 (2) Å	µ = 2.73 mm^−^^1^
*c* = 9.604 (2) Å	*T* = 113 K
β = 102.329 (4)°	Prism, colorless
*V* = 1502.8 (6) Å^3^	0.26 × 0.22 × 0.20 mm
*Z* = 4	

### Data collection

**Table:**

Rigaku Saturn CCD area-detector diffractometer	3588 independent reflections
Radiation source: rotating anode	2581 reflections with *I* > 2σ(*I*)
multilayer	*R*_int_ = 0.033
Detector resolution: 14.63 pixels mm^-1^	θ_max_ = 27.9°, θ_min_ = 2.3°
ω and φ scans	*h* = −18→18
Absorption correction: multi-scan (*CrystalClear*; Rigaku, 2007)	*k* = −14→11
*T*_min_ = 0.537, *T*_max_ = 0.611	*l* = −12→12
13836 measured reflections	

### Refinement

**Table:**

Refinement on *F*^2^	Primary atom site location: structure-invariant direct methods
Least-squares matrix: full	Secondary atom site location: difference Fourier map
*R*[*F*^2^ > 2σ(*F*^2^)] = 0.024	Hydrogen site location: inferred from neighbouring sites
*wR*(*F*^2^) = 0.058	H-atom parameters constrained
*S* = 1.04	*w* = 1/[σ^2^(*F*_o_^2^) + (0.026*P*)^2^] where *P* = (*F*_o_^2^ + 2*F*_c_^2^)/3
3588 reflections	(Δ/σ)_max_ = 0.001
174 parameters	Δρ_max_ = 0.52 e Å^−^^3^
0 restraints	Δρ_min_ = −0.28 e Å^−^^3^

### Special details

**Table:**

Geometry. All e.s.d.'s (except the e.s.d. in the dihedral angle between two l.s. planes) are estimated using the full covariance matrix. The cell e.s.d.'s are taken into account individually in the estimation of e.s.d.'s in distances, angles and torsion angles; correlations between e.s.d.'s in cell parameters are only used when they are defined by crystal symmetry. An approximate (isotropic) treatment of cell e.s.d.'s is used for estimating e.s.d.'s involving l.s. planes.
Refinement. Refinement of *F*^2^ against ALL reflections. The weighted *R*-factor *wR* and goodness of fit *S* are based on *F*^2^, conventional *R*-factors *R* are based on *F*, with *F* set to zero for negative *F*^2^. The threshold expression of *F*^2^ > σ(*F*^2^) is used only for calculating *R*-factors(gt) *etc*. and is not relevant to the choice of reflections for refinement. *R*-factors based on *F*^2^ are statistically about twice as large as those based on *F*, and *R*- factors based on ALL data will be even larger.

### Fractional atomic coordinates and isotropic or equivalent isotropic displacement parameters (Å^2^)

**Table:**

	*x*	*y*	*z*	*U*_iso_*/*U*_eq_	
Br1	0.359389 (12)	1.039199 (15)	0.078211 (19)	0.02887 (7)	
O1	0.67370 (8)	1.14977 (9)	0.58965 (11)	0.0236 (3)	
O2	0.71492 (8)	0.90082 (11)	0.30034 (13)	0.0339 (3)	
C1	0.66083 (12)	1.24071 (14)	0.68801 (17)	0.0272 (4)	
H1A	0.6506	1.3170	0.6382	0.041*	
H1B	0.7184	1.2455	0.7650	0.041*	
H1C	0.6047	1.2219	0.7281	0.041*	
C2	0.59954 (11)	1.12527 (13)	0.47931 (16)	0.0176 (3)	
C3	0.51379 (11)	1.19027 (13)	0.45009 (17)	0.0202 (4)	
H3	0.5046	1.2526	0.5124	0.024*	
C4	0.44236 (11)	1.16512 (13)	0.33205 (17)	0.0212 (4)	
H4	0.3845	1.2102	0.3123	0.025*	
C5	0.45613 (11)	1.07334 (14)	0.24293 (17)	0.0193 (4)	
C6	0.54008 (11)	1.00793 (14)	0.26967 (17)	0.0181 (3)	
H6	0.5482	0.9459	0.2063	0.022*	
C7	0.61305 (11)	1.03146 (12)	0.38797 (16)	0.0160 (3)	
C8	0.70157 (11)	0.95429 (13)	0.40477 (17)	0.0192 (3)	
C9	0.77081 (11)	0.93956 (12)	0.54697 (17)	0.0170 (3)	
H9	0.7377	0.9641	0.6244	0.020*	
C10	0.80398 (11)	0.81086 (13)	0.57217 (17)	0.0205 (4)	
H10A	0.7474	0.7589	0.5690	0.025*	
H10B	0.8367	0.7853	0.4960	0.025*	
C11	0.87275 (11)	0.79932 (13)	0.71633 (17)	0.0208 (4)	
H11A	0.8930	0.7155	0.7317	0.025*	
H11B	0.8386	0.8217	0.7921	0.025*	
C12	0.96177 (11)	0.87687 (13)	0.72848 (17)	0.0205 (4)	
H12	0.9971	0.8502	0.6545	0.025*	
C13	0.93087 (11)	1.00610 (14)	0.69645 (18)	0.0232 (4)	
H13A	0.9005	1.0362	0.7731	0.028*	
H13B	0.9887	1.0550	0.6959	0.028*	
C14	0.86004 (10)	1.01921 (14)	0.55324 (17)	0.0205 (4)	
H14A	0.8924	0.9971	0.4753	0.025*	
H14B	0.8394	1.1030	0.5393	0.025*	
C15	1.02894 (11)	0.86209 (15)	0.87409 (18)	0.0268 (4)	
H15A	0.9958	0.8927	0.9476	0.032*	
H15B	1.0405	0.7763	0.8923	0.032*	
C16	1.12570 (12)	0.92389 (19)	0.8919 (2)	0.0430 (5)	
H16A	1.1575	0.8988	0.8157	0.064*	
H16B	1.1662	0.9029	0.9846	0.064*	
H16C	1.1159	1.0099	0.8870	0.064*	

### Atomic displacement parameters (Å^2^)

**Table:**

	*U*^11^	*U*^22^	*U*^33^	*U*^12^	*U*^13^	*U*^23^
Br1	0.02108 (10)	0.03198 (11)	0.02822 (11)	0.00216 (7)	−0.00667 (7)	0.00102 (8)
O1	0.0236 (6)	0.0242 (6)	0.0208 (6)	0.0053 (5)	−0.0002 (5)	−0.0083 (5)
O2	0.0284 (7)	0.0513 (8)	0.0192 (7)	0.0171 (6)	−0.0011 (6)	−0.0109 (6)
C1	0.0309 (10)	0.0257 (9)	0.0242 (9)	0.0016 (8)	0.0045 (8)	−0.0107 (8)
C2	0.0187 (8)	0.0196 (8)	0.0147 (8)	−0.0011 (6)	0.0039 (7)	0.0027 (7)
C3	0.0238 (9)	0.0182 (8)	0.0206 (9)	0.0022 (7)	0.0094 (7)	0.0016 (7)
C4	0.0177 (8)	0.0209 (9)	0.0265 (9)	0.0031 (6)	0.0081 (7)	0.0066 (7)
C5	0.0168 (8)	0.0215 (8)	0.0178 (8)	−0.0019 (6)	−0.0002 (7)	0.0053 (7)
C6	0.0209 (9)	0.0173 (8)	0.0162 (8)	−0.0005 (6)	0.0040 (7)	0.0027 (6)
C7	0.0159 (7)	0.0164 (8)	0.0162 (8)	−0.0005 (6)	0.0044 (6)	0.0034 (7)
C8	0.0164 (8)	0.0219 (8)	0.0188 (8)	0.0005 (6)	0.0028 (7)	−0.0019 (7)
C9	0.0151 (8)	0.0199 (8)	0.0157 (8)	−0.0003 (6)	0.0027 (6)	−0.0017 (7)
C10	0.0175 (8)	0.0192 (8)	0.0240 (9)	−0.0001 (6)	0.0029 (7)	−0.0020 (7)
C11	0.0215 (8)	0.0162 (8)	0.0235 (9)	0.0013 (6)	0.0024 (7)	0.0014 (7)
C12	0.0169 (8)	0.0229 (9)	0.0202 (9)	0.0017 (6)	0.0007 (7)	−0.0016 (7)
C13	0.0180 (9)	0.0205 (8)	0.0283 (10)	−0.0041 (7)	−0.0011 (8)	0.0038 (7)
C14	0.0178 (8)	0.0208 (9)	0.0225 (9)	−0.0016 (6)	0.0036 (7)	0.0036 (7)
C15	0.0244 (9)	0.0262 (9)	0.0259 (10)	0.0017 (7)	−0.0035 (8)	0.0000 (8)
C16	0.0230 (10)	0.0583 (13)	0.0406 (13)	−0.0021 (9)	−0.0089 (9)	0.0032 (10)

### Geometric parameters (Å, °)

**Table:**

Br1—C5	1.9002 (16)	C10—C11	1.520 (2)
O1—C2	1.3536 (17)	C10—H10A	0.9900
O1—C1	1.4322 (17)	C10—H10B	0.9900
O2—C8	1.2192 (18)	C11—C12	1.521 (2)
C1—H1A	0.9800	C11—H11A	0.9900
C1—H1B	0.9800	C11—H11B	0.9900
C1—H1C	0.9800	C12—C15	1.524 (2)
C2—C3	1.398 (2)	C12—C13	1.534 (2)
C2—C7	1.413 (2)	C12—H12	1.0000
C3—C4	1.380 (2)	C13—C14	1.528 (2)
C3—H3	0.9500	C13—H13A	0.9900
C4—C5	1.383 (2)	C13—H13B	0.9900
C4—H4	0.9500	C14—H14A	0.9900
C5—C6	1.379 (2)	C14—H14B	0.9900
C6—C7	1.390 (2)	C15—C16	1.518 (2)
C6—H6	0.9500	C15—H15A	0.9900
C7—C8	1.509 (2)	C15—H15B	0.9900
C8—C9	1.512 (2)	C16—H16A	0.9800
C9—C10	1.529 (2)	C16—H16B	0.9800
C9—C14	1.544 (2)	C16—H16C	0.9800
C9—H9	1.0000		
			
C2—O1—C1	118.36 (12)	C9—C10—H10B	109.7
O1—C1—H1A	109.5	H10A—C10—H10B	108.2
O1—C1—H1B	109.5	C10—C11—C12	112.50 (13)
H1A—C1—H1B	109.5	C10—C11—H11A	109.1
O1—C1—H1C	109.5	C12—C11—H11A	109.1
H1A—C1—H1C	109.5	C10—C11—H11B	109.1
H1B—C1—H1C	109.5	C12—C11—H11B	109.1
O1—C2—C3	123.32 (14)	H11A—C11—H11B	107.8
O1—C2—C7	116.91 (14)	C11—C12—C15	110.78 (13)
C3—C2—C7	119.73 (15)	C11—C12—C13	109.46 (12)
C4—C3—C2	120.91 (15)	C15—C12—C13	112.58 (13)
C4—C3—H3	119.5	C11—C12—H12	108.0
C2—C3—H3	119.5	C15—C12—H12	108.0
C3—C4—C5	119.06 (15)	C13—C12—H12	108.0
C3—C4—H4	120.5	C14—C13—C12	112.18 (13)
C5—C4—H4	120.5	C14—C13—H13A	109.2
C6—C5—C4	121.06 (15)	C12—C13—H13A	109.2
C6—C5—Br1	119.32 (12)	C14—C13—H13B	109.2
C4—C5—Br1	119.61 (12)	C12—C13—H13B	109.2
C5—C6—C7	120.96 (15)	H13A—C13—H13B	107.9
C5—C6—H6	119.5	C13—C14—C9	110.86 (13)
C7—C6—H6	119.5	C13—C14—H14A	109.5
C6—C7—C2	118.28 (14)	C9—C14—H14A	109.5
C6—C7—C8	115.86 (14)	C13—C14—H14B	109.5
C2—C7—C8	125.85 (14)	C9—C14—H14B	109.5
O2—C8—C7	118.01 (14)	H14A—C14—H14B	108.1
O2—C8—C9	120.22 (14)	C16—C15—C12	115.32 (15)
C7—C8—C9	121.75 (14)	C16—C15—H15A	108.4
C8—C9—C10	111.48 (13)	C12—C15—H15A	108.4
C8—C9—C14	109.97 (13)	C16—C15—H15B	108.4
C10—C9—C14	108.86 (13)	C12—C15—H15B	108.4
C8—C9—H9	108.8	H15A—C15—H15B	107.5
C10—C9—H9	108.8	C15—C16—H16A	109.5
C14—C9—H9	108.8	C15—C16—H16B	109.5
C11—C10—C9	109.99 (12)	H16A—C16—H16B	109.5
C11—C10—H10A	109.7	C15—C16—H16C	109.5
C9—C10—H10A	109.7	H16A—C16—H16C	109.5
C11—C10—H10B	109.7	H16B—C16—H16C	109.5
			
C1—O1—C2—C3	5.2 (2)	C2—C7—C8—C9	22.2 (2)
C1—O1—C2—C7	−177.20 (13)	O2—C8—C9—C10	−39.5 (2)
O1—C2—C3—C4	176.77 (14)	C7—C8—C9—C10	138.54 (14)
C7—C2—C3—C4	−0.8 (2)	O2—C8—C9—C14	81.31 (18)
C2—C3—C4—C5	0.7 (2)	C7—C8—C9—C14	−100.62 (16)
C3—C4—C5—C6	−0.6 (2)	C8—C9—C10—C11	−179.57 (13)
C3—C4—C5—Br1	−178.97 (11)	C14—C9—C10—C11	58.95 (17)
C4—C5—C6—C7	0.7 (2)	C9—C10—C11—C12	−59.46 (17)
Br1—C5—C6—C7	179.06 (11)	C10—C11—C12—C15	−179.67 (13)
C5—C6—C7—C2	−0.8 (2)	C10—C11—C12—C13	55.60 (18)
C5—C6—C7—C8	−179.56 (14)	C11—C12—C13—C14	−53.76 (18)
O1—C2—C7—C6	−176.87 (13)	C15—C12—C13—C14	−177.44 (14)
C3—C2—C7—C6	0.9 (2)	C12—C13—C14—C9	56.16 (18)
O1—C2—C7—C8	1.7 (2)	C8—C9—C14—C13	179.77 (13)
C3—C2—C7—C8	179.47 (14)	C10—C9—C14—C13	−57.84 (17)
C6—C7—C8—O2	18.9 (2)	C11—C12—C15—C16	172.94 (15)
C2—C7—C8—O2	−159.73 (16)	C13—C12—C15—C16	−64.1 (2)
C6—C7—C8—C9	−159.20 (14)		
